# Secretion and Endocytosis in Pollen Tubes: Models of Tip Growth in the Spot Light

**DOI:** 10.3389/fpls.2017.00154

**Published:** 2017-02-07

**Authors:** Gleb Grebnev, Maria Ntefidou, Benedikt Kost

**Affiliations:** Cell Biology Division, Department of Biology, University of Erlangen-NurembergErlangen, Germany

**Keywords:** pollen tube, tip growth, polarized growth, secretion/exocytosis, endocytosis, membrane recycling

## Abstract

Pollen tube tip growth is a widely used model ideally suited to study cellular processes underlying polarized cell expansion. Local secretion supplying material for plasma membrane (PM) and cell wall extension is essential for this process. Cell wall biogenesis requires fusion of secretory vesicles with the PM at an about 10× higher rate than PM extension. Excess material is therefore incorporated into the PM, which needs to be reinternalized through endocytosis. The classical model of tip growth proposes that exocytosis occurs at the apex and that newly incorporated PM material is transported to adjacent lateral regions, where excess material is endocytically recycled. This model was recently challenged based on studies indicating that lateral exocytosis may be balanced by apical endocytosis. This review provides an overview of published data pertaining to exocytosis, endocytosis and vesicular trafficking in pollen tubes. Its key aim is to present classical and alternative models of tip growth in the light of available experimental data. By necessity, the review focusses on pollen tubes of angiosperm models (*Nicotiana tabacum, Arabidopsis, Lilium longiflorum*), which have been studied far more extensively and grow much faster than structurally strikingly different gymnosperm pollen tubes. Only major transport pathways are considered, which substantially contribute to the mass-flow of membrane material at the pollen tube tip. Growth oscillation, which may be displayed in particular by fast-growing pollen tubes, are not discussed as their influence on the spatial organization of apical membrane traffic is not understood.

## Pollen Tube Tip Growth is Driven By Secretion of Golgi-Derived Cell Wall Material (I.E., Pectins)

**FIGURE 1:**
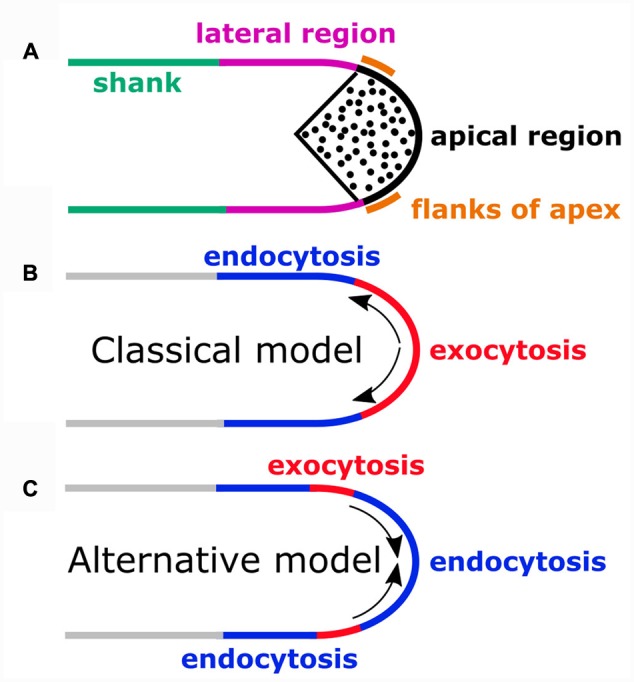
**Schematic representation of sites of exocytosis or endocytosis in different regions of the pollen tube plasma membrane (PM). (A)** The apical region of the pollen tube PM is in direct contact with the underlying cytoplasmic inverted-cone shaped region of vesicle (black dots) accumulation. In the lateral PM region, markers for clathrin mediated endocytosis (CME) have been shown to accumulate. The rest of the PM constitutes the shank region. Pollen tubes of most species have a diameter of 10–20 μm. Apical vesicles are not drawn to scale. **(B)** According to the classical model, secretion occurs apically (red PM region) and is compensated by lateral endocytosis (blue PM region). Arrows indicate retrograde transport of PM material between these sites. **(C)** The alternative model proposes compensation of lateral secretion (red PM region) by apical endocytosis (blue region at the tip). Arrows indicate anterograde transport of PM material between these sites. An additional site of endocytosis is suggested to be located laterally (blue region adjacent to the shank).

Pollen tubes have a crucial function in the fertilization of higher plants during which they act as a conduit to deliver genetic material from the site of pollination through the transmitting tract of the style to ovules. Formed as a highly elongated outgrowth upon germination of a pollen grain, pollen tubes rapidly expand exclusively in one direction. This extreme form of polarized cell expansion, which is commonly called tip growth, is based on massive secretion taking place at the tip (**Figure 1**; Steer and Steer, 1989). Golgi-derived secretory vesicles are supplying cell wall material carried in their lumen (mostly pectins that are synthesized in the Golgi) as well as material for plasma membrane (PM) extension to the site of growth. These vesicles are delivered to the tip via cytoplasmic streaming (Iwanami, 1956), which depends on actin filaments that are oriented parallel to the pollen tube long-axis (Cheung et al., 2008).

## Pectin Distribution and Modification in the Pollen Tube Cell Wall

The apical dome is the only region of the pollen tube cell wall that undergoes growth (Rosen, 1961). Therefore, this region has a requirement for both plasticity to allow apical cell expansion and sufficient stiffness to withstand turgor pressure to prevent cell bursting. The cell wall is primarily made of secreted esterified pectins at the apex, whereas laterally and in the shank it contains de-esterified pectins along with callose and cellulose, which are not secreted but synthesized by transmembrane enzyme complexes integrated into the PM (Chebli et al., 2012). Esterified pectins carried in the lumen of Golgi-derived secretory vesicles (Sterling et al., 2001) are deposited in the cell wall at the tip when these vesicles fuse with the PM. Differential distribution of esterified and de-esterified pectins can be visualized by immunofluorescence using the monoclonal antibodies JIM7 and JIM5, respectively (Knox et al., 1990). Esterified pectins detected by the JIM7 antibody are predominantly found in pollen tube cell wall at the apex, while de-esterified pectins detected by JIM5 antibody are present in the cell wall in the later region and in the shank (Bosch and Hepler, 2005; Bosch et al., 2005; Parre and Geitmann, 2005; Chebli et al., 2012). Pectin de-esterification along with cellulose and callose deposition enhances the mechanical stability of the pollen tube cell wall in the lateral region and in the shank such that these cell wall regions don’t expand in response to turgor pressure and the cylindrical shape of pollen tubes is maintained. Pectin methylesterases (PMEs; Micheli, 2001), secreted cell wall associated enzymes, are responsible for pectin de-esterification, which results in the generation of free, deprotonated carboxyl groups that can be cross-linked by calcium to generate stiff pectate gels (Bosch and Hepler, 2005).

Pectin methylesterases responsible for pectin de-esterification as well as PME inhibitors (PMEIs; Giovane et al., 2004), which regulate PME activity, are expressed at high levels in pollen (Bosch et al., 2005) and pollen tubes (Bosch and Hepler, 2005). Two types of PMEs are known to exist. Type I PMEs contain a pro-region that is lacking in type II PMEs. The pro-region of type I PMEs is thought to autoinhibit PME activity not only during the co-transport of these proteins together with esterified pectins within the lumen of secretory vesicles to the site of exocytosis (Micheli, 2001), but possibly also within the apical cell wall after secretion. Similarly, PMEIs appear to inhibit the PME activity of type II PMEs that lack an autoinhibitory pro-region (Röckel et al., 2008). In fact, AtPPME1, a type II PME, was shown to bind to the PMEIs AtPMEI1 and AtPMEI2 *in vitro* and to be inactivated by AtPMEI2 when co-expressed with this protein in *Nicotiana benthamiana* leaves (Röckel et al., 2008).

Consistent with the observed distribution of esterified and de-esterified pectins within the pollen tube cell wall, transient expression of YFP-tagged AtPMEI1 and AtPMEI2 revealed that these PMEIs exclusively accumulate in the apical cell wall of *N. tabacum* pollen tubes, whereas YFP-tagged AtPPME1 uniformly labeled the entire cell wall (Röckel et al., 2008). In fact, the lateral cell wall region in which the fluorescence intensity of YFP-tagged PMEIs sharply declined exactly coincided with the border between the accumulation of esterified pectins at the apex and of de-esterified pectins laterally as well as in the shank. Interestingly, AtPMEI2 was detected in Brefeldin A-induced aggregates within the cytoplasm of *N. tabacum* pollen tubes suggesting that this cell wall protein undergoes endocytic recycling (Röckel et al., 2008). Lateral endocytic internalization of AtPMEI2 was therefore proposed to be responsible for restricting the localization of this protein to the cell wall at the pollen tube apex (Röckel et al., 2008).

## Classical Model of Tip Growth

As strongly suggested by the apical accumulation of secreted esterified pectins and PMEIs, the classical model of tip growth (**Figure 1B**; Steer and Steer, 1989; Derksen et al., 1995; Kost, 2008) proposes that exocytosis is focused on the pollen tube apex. According to this model, to provide sufficient material for cell wall biogenesis, secretory vesicles need to fuse with the apical PM at a substantially higher rate than is required for PM extension (Picton and Steer, 1983; Derksen et al., 1995; Bove et al., 2008; Ketelaar et al., 2008). This results in the incorporation of excess material into the apical pollen tube PM, which needs to be recycled via lateral endocytosis (Derksen et al., 1995). Delivery of excess membrane material to the apex, which is compensated by lateral endocytic internalization, was postulated to result in constant retrograde transport of PM components from the apical site of secretion to the lateral site of endocytic internalization (**Figure 1B**; Kost, 2008). In the following three chapters, further experimental evidence in addition to the results of the analysis of pectin and PMEI distribution in pollen tubes is described, which is consistent with the classical model.

## Apical Secretion in Pollen Tubes

The classical model of tip growth originated from the analysis of transmission electron micrographs depicting a stunning zonation of the pollen tube cytoplasm, which was found to be exclusively populated by vesicles in an inverted cone-shaped region at the apex (**Figure 1A**; Rosen et al., 1964; Rosen and Gawlik, 1965; Tiwari and Polito, 1988; Lancelle and Hepler, 1992; Derksen et al., 1995). The accumulation of these vesicles, which were identified as secretory vesicles (Lancelle and Hepler, 1992; Derksen et al., 1995), in direct contact with the apical PM provided indirect evidence suggesting that secretion occurs at the apex. Consistent with this interpretation, vesicles in the inverted cone-shaped region were found to be of similar electron density as the apical cell wall (Lancelle and Hepler, 1992; Derksen et al., 1995) and were occasionally observed on transmission electron micrographs to fuse with the apical PM (Steer and Steer, 1989; Lancelle and Hepler, 1992). Further indication of apical secretion was provided by Picton and Steer (1983) who employed culture media containing Ca^2+^ at different concentrations to grow *Tradescantia virginiana* pollen tubes at varying rates. Slowly growing pollen tubes were found to display increased cell wall thickness at the apex, whereas all regions of the cell wall of rapidly growing pollen tubes were of equally thickness (Picton and Steer, 1983). This observation suggests that at slow growth rates excess material not required for expansion is deposited in the cell wall by apical secretion.

More recently, direct evidence for apical secretion in pollen tubes was generated using live-cell fluorescence microscopy. To locate the site of secretion in *N. tabacum* pollen tubes, Lee et al. (2008) studied the dynamics of the incorporation of a GFP-tagged transmembrane protein, the receptor-like kinase AtPRK1 (AtPRK1::GFP), into the apical PM based on fluorescence recovery after photobleaching (FRAP; Axelrod et al., 1976; Lippincott-Schwartz et al., 2003). Treatment with Brefeldin A, an inhibitor of secretion (Nebenführ et al., 2002), disrupted PM labeling by AtPRK1::GFP and arrested this fusion protein in the endoplasmic reticulum (ER), which was identified based on co-labeling with a specific fluorescent marker. This confirmed that AtPRK1::GFP as expected is inserted into PM via exocytosis. To identify the exact site of secretion, Lee et al. (2008) selectively bleached AtPRK1::GFP in the PM either at the apex or in an adjacent lateral region. Rapid fluorescence recovery was observed at the apex, demonstrating that secretion occurs in this region. Detailed analysis showed that fluorescence first recovered in the center of the apex and gradually spread from there to the flanks of apex. By contrast, no fluorescence recovery was observed after bleaching lateral PM regions, indicating that these regions are devoid of detectable exocytic activity. These results were recently confirmed by the same laboratory using AtPRK1 fused to the photoconvertible fluorescent protein Dendra2 (Luo et al., 2016). Photoconverted AtPRK1::Dendra2 also first appeared in the center of the apex and spread distally from this site. In addition, AtPRK1::Dendra2 enabled measurement of local exocytosis rates, which were determined to be highest in the center of the apex and twofold lower at its flanks (Luo et al., 2016).

Similar experiments were also performed using GFP fused to the pollen-specific *N. tabacum* PME, NtPPME1. NtPPME1::GFP exclusively labeled the apical cell wall of *N. tabacum* pollen tubes (Bosch et al., 2005; Wang et al., 2013). Application of FRAP methodology established that after bleaching the apical cell wall recovery of NtPPME1::GFP fluorescence also first occurred in the center of the apex, from where it spread distally (Wang et al., 2013). Together, the observations summarized in this chapter strongly suggest that secretion delivering transmembrane proteins to the PM as well as extracellular proteins to the cell wall is focused on the pollen tube apex.

## Endocytosis in Pollen Tubes and Need for Membrane Recycling

The first indication that not only secretion but also endocytosis occurs during pollen tube tip growth came from studies, which quantitatively compared the amount of membrane material required for PM extension to the amount of such material actually supplied by secretory vesicles. As discussed in detail in the next two paragraphs, it was determined that fusion of secretory vesicles with the apical PM provided significantly more membrane material than required for PM extension (Picton and Steer, 1983; Steer and Steer, 1989; Derksen et al., 1995; Bove et al., 2008; Ketelaar et al., 2008), suggesting that maintenance of pollen tube tip architecture requires internalization of excess membrane material by endocytosis (Steer, 1988). Furthermore, local incorporation of excess membrane material into the PM, which is compensated by endocytic internalization at a different site, was postulated to drive active transport of membrane material within the PM from the site of secretion to the site of endocytosis (Kost, 2008; Zonia and Munnik, 2008, 2009).

As discussed in the previous chapter, Picton and Steer (1983) found that growing *T. virginiana* pollen tubes at reduced rates in culture medium containing low levels of Ca^2+^ resulted in a substantial thickening of the apical cell wall. This observation led to the conclusion that irrespective of the speed of tip growth, constant numbers of secretory vesicles are produced by the Golgi apparatus and subsequently fuse with the PM. Furthermore, the authors of this study determined that in slowly growing pollen tubes at least 80% of the membrane material delivered to the PM by secretion is not required for the extension and needs to be internalized by endocytosis.

Derksen et al. (1995) used electron micrographs of *N. tabacum* pollen tubes grown at normal rates to measure the average volume of individual secretory vesicles as well as of the apical cell wall. Based on these data they were able to determine the rate at which secretory vesicles need to fuse with PM to provide sufficient material for cell wall extension. Interestingly, they found that at this rate, secretion results in the incorporation of about 10× more membrane material into the PM than required for the extension of this structure. Consequently, during *N. tabacum* pollen tube tip growth at normal rates, roughly 90% of the membrane material delivered to the PM by secretion needs to be reinternalized by endocytosis. Based on similar approaches, secretion was also estimated to deliver about 80% excess membrane material destined for endocytic internalization in normally growing *Arabidopsis* (Ketelaar et al., 2008) and *Lilium longiflorum* (Bove et al., 2008) pollen tubes. These estimations are obviously based on the assumption that secretory vesicles completely fuse with the pollen tube PM rather than delivering their cargo based on temporary “kiss-and-run” fusion. Analysis of the fusion of FM4-64 labeled secretory vesicles with the PM in gymnosperm (*Picea meyeri*) pollen tubes using evanescent wave microscopy in fact strongly supported complete incorporation and did not provided any evidence for kiss-and-run temporary fusion (Wang et al., 2006). However, similar experiments also need to be performed with angiosperm pollen tubes, which grow much faster than gymnosperm pollen tubes and are structurally substantially different (Fernando et al., 2005).

Direct evidence of endocytosis in pollen tubes was generated after fluorescently labeled dextrans became available, which plant cells were demonstrated to endocytically internalize and accumulate in their vacuoles (Cole et al., 1990). Growing pollen tubes of different species were shown to endocytose fluorescent dextrans added to their culture medium (Hann, 1991; O’Driscoll et al., 1993). Time lapse experiments showed that internalized fluorescent dextrans first label small vacuoles in the cytoplasm near the pollen tube tip before they are transported to the main central vacuole in the shank (O’Driscoll et al., 1993). However, these experiments did not provide insights into the exact site of endocytic uptake at the PM.

Later, endocytosis in growing pollen tubes was confirmed by the application of FM4-64, a fluorescent styryl dye that labels membranes and serves as a tracer of endocytic membrane uptake and trafficking (Fischer-Parton et al., 2000). Time-lapse experiments typically show that FM4-64 initially exclusively labels the PM of various cell types, before it is transported via early and late endosomes to the vacuole (Vida and Emr, 1995; Fischer-Parton et al., 2000). Interestingly, staining of *L. longiflorum* and *N. tabacum* pollen tubes with FM4-64 revealed that this dye at early time-points after application (15 min) strongly accumulates in the vesicle rich inverted cone-shaped region at the apex, before it can eventually (after 12 h) be detected in vacuoles (Parton et al., 2001). This observation prompted Parton et al. (2001) to suggest that specifically in pollen tubes most of the endocytosed membrane material is rapidly recycled to the secretory pathway, whereas only a minor proportion of this material is transported along the standard route to the vacuole. Diverting the majority of the endocytosed membrane material to the recycling pathway is likely to represent an adaptation to the specific requirements of rapid pollen tube tip growth. Consistent with this hypothesis, we have recently found that in *N. tabacum* pollen tubes a special compartment of the *trans*-golgi network (TGN), which in plant cells not only generates secretory vesicles but also serves as early endosome (Dettmer et al., 2006), is located right at the interface between the inverted cone-shaped region of vesicle accumulation at the apex and the adjacent regular cytoplasm (Stephan et al., 2014). Obviously this TGN compartment is ideally positioned to integrate exocytic and endocytic membrane trafficking pathways at the pollen tube tip und to mediate the massive recycling of endocytosed membrane material to the secretory pathway that appears to be required for tip growth.

## Lateral Endocytosis in Pollen Tubes

Transmission electron microscopy of *N. tabacum* pollen tubes revealed that 50% of all coated pits observed at the PM, which are sites of clathrin mediated endocytosis (CME), were concentrated in a lateral region spanning from 6 to 15 μm behind the extreme apex (Derksen et al., 1995). The identification of the PM in this zone as a major site of endocytic membrane internalization was later corroborated by immunofluorescence and fluorescent protein tagging experiments, which showed that important CME markers such as clathrin heavy chain (CHC), clathrin light chain (CLC) and AP180 accumulate at the PM in the same region. More specifically, Blackbourn and Jackson (1996) detected CHC both at the PM and in the cytoplasm of *L. longiflorum* pollen tubes based on immunofluorescence analysis. Biochemical analysis showed that a large proportion of the CHC present in these cells is in the assembled state indicating high endocytic activity. Interestingly, CHC was found to accumulate at the PM not only laterally but also at the apex, although labeling of the lateral PM was significantly stronger. By contrast, in a later study employing the same methodology for CHC localization in *L. longiflorum* pollen tubes, Zhao et al. (2010) observed accumulation of this protein specifically at the lateral PM as well as at the flanks of the apex. In *N. tabacum* pollen tubes, immunofluorescence analysis of CLC localization (Feng et al., 2016) resulted in a clear punctate labeling of the lateral PM with a significantly weaker fluorescent signal also detectable at the PM at the flanks of apex. In addition, CLC was observed in the cytoplasm, where it accumulated to highest levels right beneath the lateral PM (Feng et al., 2016). GFP-tagged AP180 was also found to specifically accumulate at the lateral PM of *N. tabacum* pollen tubes (Zhao et al., 2010), essentially in the same region with which CHC and CLC are most strongly associated.

Further evidence supporting active endocytic membrane internalization specifically at the lateral PM derives from the observation of the uptake of fluorescently labeled lipids. External application of bis-BODIPY FL C_11_-phosphocholine to cultured *N. sylvestris* pollen tubes resulted in specific labeling of the cytoplasm beneath the lateral PM with which CHC was associated as confirmed by immunogold labeling and transmission electron microscopy (Lisboa et al., 2008). Externally applied phosphatidic acid labeled with BODIPY was also observed to accumulate in the cytoplasm beneath the lateral PM in *N. tabacum* pollen tubes (Potocký, 2003), although in this case a cytoplasmic region slightly further away from the apex was stained by the internalized lipid.

Additional evidence for lateral endocytosis was generated by visualizing the internalization of externally applied positively charged nanogold particles into *N. tabacum* pollen tubes using transmission electron microscopy (Moscatelli et al., 2007). These particles were described to be found at early time points within vesicles right beneath the lateral PM and at later time points in the lumen of secretory (Golgi stacks, apical vesicles) as well as late endocytic (late endosomes, vacuoles) membrane compartments. These observations are consistent with the FM4-64 transport routes described in the previous chapter and indicate that material endocytosed laterally at the pollen tube PM is either recycled to the secretory endomembrane system or transported to the vacuole for degradation. Particles also appeared to be internalized and selectively transported to the vacuole in the presence of the CME inhibitor Ikarugamycin, indicating that clathrin-independent endocytosis may also occur at the lateral pollen tube PM and specifically feed into the membrane transport pathway to the vacuole. However, this clathrin-independent pathway seems to be responsible for a minor proportion of the total endocytic membrane uptake, as Ikarugamycin almost completely blocked FM4-64 internalization (Moscatelli et al., 2007). Interestingly, consistent with active endocytosis occurring at the lateral pollen tube PM, which is enriched in coated pits and CME markers, FM4-64 specifically accumulated at this site in the presence of Ikarugamycin.

## Alternative Model of Tip Growth and Supporting Evidence

An alternative model of tip growth was postulated by Zonia and Munnik (2008, 2009) and Bove et al. (2008), which deviates from the classical model with respect to the proposed locations of sites of secretions and endocytosis (**Figure 1**). According to the alternative model (**Figure 1C**), in addition to lateral endocytosis, which is also supported by the classical model (**Figure 1B**), endocytosis also takes place at the apex while secretion is confined to a lateral region immediately adjacent to the flanks of the apex. Membrane material newly inserted into the PM at this site is proposed to enable PM extension by traveling in anterograde direction toward the apex, where excess material is endocytically internalized (**Figure 1C**; Zonia and Munnik, 2008, 2009).

Evidence for the alternative model is largely based on time-lapse fluorescence microscopic analysis of the internalization of red fluorescent FM4-64 into *N. tabacum* pollen tubes preloaded with FM1-43, a close related styryl dye that emits green fluorescence (Zonia and Munnik, 2008). Minutes after FM4-64 application, this dye appeared to co-localize with preloaded FM1-43 specifically at the interface between the apical PM and the inverted cone-shape zone of vesicle accumulation directly underneath. This observation prompted the authors to conclude that FM4-64 is endocytically internalized at the pollen tube apex. Furthermore, they proposed to have demonstrated endocytic internalization of FM4-64 also at the lateral pollen tube PM based on the detection of red fluorescent vesicles in the cytoplasm beneath this PM region shortly after FM4-64 application. However, this finding should be regarded with caution, as similar observations were considered inconclusive in previous studies (Parton et al., 2001). To support the presence of sites of secretion in a lateral region directly adjacent to the flanks of the pollen tube apex, results of time-lapse fluorescence or transmission light (differential interference contrast) microscopic imaging of styryl dye labeled or unstained pollen tubes, respectively, were presented, which according to the authors show fusion of single secretory vesicles with the lateral PM (Zonia and Munnik, 2008). Bove et al. (2008) investigated patterns of mobility of cytoplasmic components at the tip of *L. longiflorum* pollen tubes using time-lapse transmission light (differential interference contrast) microscopy as well as FRAP analysis of FM1-43 stained endomembrane compartments. Based on the observed patterns, the authors also suggested that during pollen tube tip growth, endocytosis may occur not only laterally but also apically, whereas secretion may be focused on a lateral region.

Additional evidence supporting endocytic membrane internalization at the pollen tube apex was provided by Moscatelli et al. (2007), who employed transmission electron microscopy to observe the uptake of positively or negatively charged nanogold particles into *N. tabacum* pollen tubes. By contrast to positively charged particles, which appeared to be endocytosed laterally (see previous chapter), negatively charged particles were described to be detected in the lumen of vesicles in the inverted cone-shaped zone at early time points after application, and within vacuoles at later time points. Based on these observations, the authors proposed that endocytosis is also occurring at the pollen tube apex and serves to transport material to the vacuole for degradation.

## Conclusion

A large amount of evidence is supporting the classical model of pollen tube tip growth proposing apical secretion and lateral endocytosis. However, with the introduction of an alternative model suggesting that lateral secretion may be balanced by apical endocytic membrane recycling, tip growth must be reinvestigated with respect to the exact location of sites of exocytosis and endocytosis. To this end, the dynamic behavior of membrane trafficking markers such as transmembrane proteins fused to a fluorescent protein, FM4-64 and fluorescently labeled membrane lipids needs to be further characterized in growing pollen tubes using advanced fluorescence microscopy methods (FRAP and photoactivation/photoconversion experiments) in conjunction with pharmacological analysis (application of exocytosis or endocytosis inhibitors).

## Author Contributions

BK and GG developed the concept of this review. GG wrote the text with the support of BK. MN contributed ideas and critical revision.

## Conflict of Interest Statement

The authors declare that the research was conducted in the absence of any commercial or financial relationships that could be construed as a potential conflict of interest.

The handling Editor declared a shared affiliation, though no other collaboration, with the authors and states that the process nevertheless met the standards of a fair and objective review.

## References

[B1] Axelrod D. Koppel D. E. Schlessinger J. Elso E. Webb W. W. (1976). Mobility measurement by analysis of fluorescence photobleaching recovery kinetics. *Biophys. J.* 16 1055–1069. 10.1016/S0006-3495(76)85755-4 786399 PMC1334945

[B2] Blackbourn H. D. Jackson A. P. (1996). Plant clathrin heavy chain: sequence analysis and restricted localisation in growing pollen tubes. *J. Cell Sci.* 109 777–786. 8718669 10.1242/jcs.109.4.777

[B3] Bosch M. Cheung A. Y. Hepler P. K. (2005). Pectin methylesterase, a regulator of pollen tube growth. *Plant Physiol.* 138 1334–1346. 10.1104/pp.105.059865 15951488 PMC1176407

[B4] Bosch M. Hepler P. K. (2005). Pectin methylesterases and pectin dynamics in pollen tubes. *Plant Cell* 17 3219–3226. 10.1105/tpc.105.037473 16322606 PMC1315365

[B5] Bove J. Vaillancourt B. Kroeger J. Hepler P. K. Wiseman P. W. Geitmann A. (2008). Magnitude and direction of vesicle dynamics in growing pollen tubes using spatiotemporal image correlation spectroscopy and fluorescence recovery after photobleaching. *Plant Physiol.* 147 1646–1658. 10.1104/pp.108.120212 18508956 PMC2492615

[B6] Chebli Y. Kaneda M. Zerzour R. Geitmann A. (2012). The cell wall of the *Arabidopsis* pollen tube–spatial distribution, recycling, and network formation of polysaccharides. *Plant Physiol.* 160 1940–1955. 10.1104/pp.112.199729 23037507 PMC3510122

[B7] Cheung A. Y. Duan Q. H. Costa S. S. de Graaf B. H. Di Stilio V. S. Feijo J. (2008). The dynamic pollen tube cytoskeleton: live cell studies using actin-binding and microtubule-binding reporter proteins. *Mol. Plant.* 1 686–702. 10.1093/mp/ssn026 19825573

[B8] Cole L. Coleman J. Evans D. Hawes C. (1990). Internalisation of fluorescein isothiocyanate-dextran by suspension-cultured plant cells. *J. Cell Sci.* 96 721–730.

[B9] Derksen J. Rutten T. Lichtscheidl I. K. de Win A. H. N. Pierson E. S. Rongen G. (1995). Quantitative analysis of the distribution of organelles in tobacco pollen tubes: implications for exocytosis and endocytosis. *Protoplasma* 188 267–276. 10.1007/BF01280379

[B10] Dettmer J. Hong-Hermesdorf A. Stierhof Y. D. Schumacher K. (2006). Vacuolar H+-ATPase activity is required for endocytic and secretory trafficking in *Arabidopsis*. *Plant Cell* 18 715–730. 10.1105/tpc.105.037978 16461582 PMC1383645

[B11] Feng Q. N. Kang H. Song S. J. Ge F. R. Zhang Y. L. Li E. (2016). *Arabidopsis* RhoGDIs are critical for cellular homeostasis of pollen tubes. *Plant Physiol.* 170 841–856. 10.1104/pp.15.01600 26662604 PMC4734571

[B12] Fernando D. D. Lazzaro M. D. Owens J. N. (2005). Growth and development of conifer pollen tubes. *Sex. Plant Reprod.* 18 149–162. 10.1007/s00497.005.0008.y

[B13] Fischer-Parton S. Parton R. M. Hickey P. C. Dijksterhuis J. Atkinson H. A. Read N. D. (2000). Confocal microscopy of FM4-64 as a tool for analysing endocytosis and vesicle trafficking in living fungal hyphae. *J. Microsc.* 198 246–259. 10.1046/j.1365-2818.2000.00708.x 10849201

[B14] Giovane A. Servillo L. Balestrieri C. Raiola A. D’Avino R. Tamburrini M. (2004). Pectin methylesterase inhibitor. *Biochim. Biophys. Acta* 1696 245–252. 10.1016/j.bbapap.2003.08.011 14871665

[B15] Hann C. (1991). *Uptake of Fluorescent Dextran by Endocytosis in Pollen Tubes of Tradescantia Virginiana and in Cells of Morinda citrifolia.* Ph.D. thesis, University College Dublin, Dublin.

[B16] Iwanami Y. (1956). Protoplasmic movement in pollen grains and tubes. *Phytomorphol* 6 288–295.

[B17] Ketelaar T. Galway M. E. Mulder B. M. Emons A. M. (2008). Rates of exocytosis and endocytosis in *Arabidopsis* root hairs and pollen tubes. *J. Microsc.* 231 265–273. 10.1111/j.1365-2818.2008.02031.x 18778424

[B18] Knox J. P. Linstead P. J. King J. Cooper C. Roberts K. (1990). Pectin esterification is spatially regulated both within cell walls and between developing tissues of root apices. *Planta* 181 512–521. 10.1007/BF00193004 24196931

[B19] Kost B. (2008). Spatial control of Rho (Rac-Rop) signaling in tip-growing plant cells. *Trends Cell Biol.* 18 119–127. 10.1016/j.tcb.2008.01.003 18280158

[B20] Lancelle S. A. Hepler P. K. (1992). Ultrastructure of freeze-substituted pollen tubes of Lilium longiflorum. *Protoplasma* 167 215–230. 10.1007/BF01403385

[B21] Lee Y. J. Szumlanski A. Nielsen E. Yang Z. (2008). Rho-GTPase–dependent filamentous actin dynamics coordinate vesicle targeting and exocytosis during tip growth. *J. Cell Biol.* 181 1155–1168. 10.1083/jcb.200801086 18591430 PMC2442199

[B22] Lippincott-Schwartz J. Altan-Bonnet N. Patterson G. H. (2003). Photobleaching and photoactivation: following protein dynamics in living cells. *Nat. Cell Biol.* 5 S7–S14. 10.1038/ncb1032 14562845

[B23] Lisboa S. Scherer G. E. Quader H. (2008). Localized endocytosis in tobacco pollen tubes: visualisation and dynamics of membrane retrieval by a fluorescent phospholipid. *Plant Cell Rep.* 27 21–28. 10.1007/s00299-007-0437-1 17786450

[B24] Luo N. Yan A. Yang Z. (2016). Measuring Exocytosis Rate Using Corrected Fluorescence Recovery After Photoconversion. *Traffic* 17 554–564. 10.1111/tra.12380 26822068 PMC7513930

[B25] Micheli F. (2001). Pectin methylesterases: cell wall enzymes with important roles in plant physiology. *Trends Plant Sci.* 6 414–419. 10.1016/S1360-1385(01)02045-3 11544130

[B26] Moscatelli A. Ciampolini F. Rodighiero S. Onelli E. Cresti M. Santo N. (2007). Distinct endocytic pathways identified in tobacco pollen tubes using charged nanogold. *J. Cell Sci.* 120 3804–3819. 10.1242/jcs.012138 17940063

[B27] Nebenführ A. Ritzenthaler C. Robinson D. G. (2002). Brefeldin a: deciphering an enigmatic inhibitor of secretion. *Plant Physiol.* 130 1102–1108. 10.1104/pp.011569 12427977 PMC1540261

[B28] O’Driscoll D. Hann C. Read S. M. Steer M. W. (1993). Endocytotic uptake of fluorescent dextrans by pollen tubes grown in vitro. *Protoplasma* 175 126–130. 10.1007/BF01385010

[B29] Parre E. Geitmann A. (2005). Pectin and the role of the physical properties of the cell wall in pollen tube growth of *Solanum chacoense*. *Planta* 220 582–592. 10.1007/s00425-004-1368-5 15449057

[B30] Parton R. M. Fischer-Parton S. Watahiki M. K. Trewavas A. J. (2001). Dynamics of the apical vesicle accumulation and the rate of growth are related in individual pollen tubes. *J. Cell Sci.* 114 2685–2695. 11683395 10.1242/jcs.114.14.2685

[B31] Picton J. M. Steer M. W. (1983). Membrane recycling and the control of secretory activity in pollen tubes. *J. Cell Sci.* 63 303–310. 6630312 10.1242/jcs.63.1.303

[B32] Potocký M. Eliás M. Profotová B. Novotná Z. Valentová O. Zárský V. (2003). Phosphatidic acid produced by phospholipase D is required for tobacco pollen tube growth. *Planta* 217 122–130. 12721856 10.1007/s00425-002-0965-4

[B33] Röckel N. Wolf S. Kost B. Rausch T. Greiner S. (2008). Elaborate spatial patterning of cell-wall PME and PMEI at the pollen tube tip involves PMEI endocytosis, and reflects the distribution of esterified and de-esterified pectins. *Plant J.* 51 133–143. 10.1111/j.1365-313X.2007.03325.x 17971035

[B34] Rosen W. G. (1961). Studies of pollen tube chemotropism. *Am. J. Bot.* 481 889–895. 10.2307/2439530

[B35] Rosen W. G. Gawlik S. R. (1965). Fine structure of Lily pollen tubes following various fixation and staining procedures. *Protoplasma* 61 181–191. 10.1007/BF01247918 4162185

[B36] Rosen W. G. Gawlik S. R. Dashek W. V. Siegesmund K. A. (1964). Fine structure and cytochemistry of lilium pollen tubes. *Am. J. Bot.* 51 61–71. 10.2307/2440065

[B37] Steer M. W. (1988). Plasma membrane turnover in plant cells. *J. Exp. Bot.* 39 987–996. 10.1093/jxb/39.8.987

[B38] Steer M. Steer J. (1989). Pollen tube tip growth. *New. Phytol.* 111 323–358. 10.1111/j.1469-8137.1989.tb00697.x 33874021

[B39] Stephan O. Cottier S. Fahlen S. Montes-Rodriguez A. Sun J. Eklund D. M. (2014). RISAP is a TGN-associated RAC5 effector regulating membrane traffic during polar cell growth in tobacco. *Plant Cell* 26 4426–4447. 10.1105/tpc.114.131078 25387880 PMC4277221

[B40] Sterling J. D. Quigley H. F. Orellana A. Mohnen D. (2001). The catalytic site of the pectin biosynthetic enzyme alpha-1,4-galacturonosyltransferase is located in the lumen of the Golgi. *Plant Physiol.* 127 360–371. 10.1104/pp.127.1.360 11553763 PMC117991

[B41] Tiwari S. C. Polito V. S. (1988). Organization of the cytoskeleton in pollen tubes of Pyrus communis: a study employing conventional and freeze-substitution electron microscopy, immunofluorescence, and rhodamine-phalloidin. *Protoplasma* 147 100–112. 10.1007/BF01403337

[B42] Vida T. A. Emr S. D. (1995). A new vital stain for visualizing vacuolar membrane dynamics and endocytosis in yeast. *J. Cell Biol.* 128 779–792. 10.1083/jcb.128.5.779 7533169 PMC2120394

[B43] Wang H. Zhuang X. Cai Y. Cheung A. Y. Jiang L. (2013). Apical F-actin-regulated exocytic targeting of NtPPME1 is essential for construction and rigidity of the pollen tube cell wall. *Plant J.* 76 367–379. 10.1111/tpj.12300 23906068

[B44] Wang X. Teng Y. Wang Q. Li X. Sheng X. Zheng M. (2006). Imaging of dynamic secretory vesicles in living pollen tubes of Picea meyeri using evanescent wave microscopy. *Plant Physiol.* 141 1591–1603. 10.1104/pp.106.080168 16798949 PMC1533916

[B45] Zhao Y. Yan A. Feijó J. A. Furutani M. Takenawa T. Hwang I. (2010). Phosphoinositides regulate clathrin-dependent endocytosis at the tip of pollen tubes in *Arabidopsis* and tobacco. *Plant Cell* 22 4031–4044. 10.1105/tpc.110.076760 21189293 PMC3027160

[B46] Zonia L. Munnik T. (2008). Vesicle trafficking dynamics and visualization of zones of exocytosis and endocytosis in tobacco pollen tubes. *J. Exp. Bot.* 59 861–873. 10.1093/jxb/ern007 18304978

[B47] Zonia L. Munnik T. (2009). Uncovering hidden treasures in pollen tube growth mechanics. *Trends Plan. Sci.* 14 318–327. 10.1016/j.tplants.2009.03.008 19446491

